# Outcomes and cost analysis of laparoscopic versus open appendectomy for treatment of acute appendicitis: 4-years experience in a district hospital

**DOI:** 10.1186/1471-2482-14-14

**Published:** 2014-03-19

**Authors:** Vincenzo Minutolo, Alessio Licciardello, Biagio Di Stefano, Manuel Arena, Goffredo Arena, Vincenzo Antonacci

**Affiliations:** 1 Department of Surgical Sciences, Organ Transplantation and Advanced Technologies, University of Catania, via Santa Sofia 84, 95123 Catania, Italy; 2 Department of Surgery, McGill University, 687 Pine Avenue, Montreal, QC H3A1A1, Canada; 3 Division of General Surgery, Civil Hospital of Ragusa, Piazza Caduti di Nassiryia 1, 97100 Ragusa, Italy

**Keywords:** Laparoscopic appendectomy, Open appendectomy, Costs, Complications, Intra-abdominal abscess, Operative time, Length of hospital stay

## Abstract

**Background:**

Laparoscopic appendectomy is not yet unanimously considered the “gold standard” in the treatment of acute appendicitis because of its higher operative time, intra-abdominal abscess risk, and costs compared to open appendectomy. This study aimed to compare outcomes and cost of laparoscopic and open appendectomy in a district hospital.

**Methods:**

A retrospective analysis of 230 patients who underwent appendectomy at the Division of General Surgery of the Civil Hospital of Ragusa, Italy, from May 2008 to May 2012 was performed. The variables analyzed included patients data (age, gender, previous abdominal surgery, preoperative WBC count, duration of symptoms, ASA risk score), rate of uncomplicated or complicated appendicitis, operative time, postoperative complications, length of hospital stay, and total costs. The patients were divided in two groups according to the surgical approach and compared for each variable. The results were analyzed using the t Student test for quantitative variables, and the Chi-square test with Yates correction and Fisher exact test for categorical.

**Results:**

Laparoscopic appendectomy was performed in 139 patients, open appendectomy in 91. Two cases (1.4%) were converted to open procedure and included in the laparoscopic group data. Patient data and rate of complicated appendicitis were similar in the two study groups. There was no statistical difference (p = 0.476) in the mean operative time between the laparoscopic (52.2 min; range, 20–155) and open appendectomy (49.3 min; range, 20–110) groups. The overall incidence of minor and major complications was significantly lower (p = 0.006) after laparoscopic appendectomy (2.9%, 4 cases) than after open appendectomy (13.2%, 12 cases); rate of intra-abdominal abscess were similar. The length of hospital stay was significantly shorter (p = 0.001) in laparoscopic group (2.75 days; range, 1–8) than in open group (3.87 days; range, 1–19). The mean total cost was 2282 Euro in laparoscopic group and 2337 Euro in open group, with a no significant difference of 55 Euro (p = 0.812).

**Conclusion:**

Laparoscopic appendectomy is associated with fewer complications, shorter hospital stay, and similar operative time, intra-abdominal abscess rate, and total costs, compared with open appendectomy. Therefore, laparoscopic appendectomy can be recommended as preferred approach in acute appendicitis.

## Background

Since its introduction into clinical practice in 1983, laparoscopic appendectomy (LA) [1] proved to be a feasible and safe procedure and has gained worldwide acceptance [2]. The clinical advantages of LA, such as reduced hospital stay, lower incidence of wound infection, faster return to normal work activities, shorter postoperative ileus, less postoperative pain and better cosmetic results have been demonstrated over the years by several studies and meta-analysis [3-9]. However, the application of LA as “gold standard” in the treatment of acute appendicitis is still debated because of longer operative time, higher risk for postoperative intra-abdominal abscesses, and higher costs, as it was described by several authors who compared LA to open appendectomy (OA) [8,10]. In particular, over the past two decades, the worldwide cuts in hospital budgets due to the economic crisis that has hit several countries, made the reduction of expenses a key factor in the choice of a particular diagnostic or therapeutic procedure, particularly in hospital settings where the budgets are limited. The aim of our study was to compare the outcomes between laparoscopic and open treatment for acute appendicitis, performed in a district hospital, in terms of operative time, rate of complications, length of hospital stay and costs.

## Methods

We performed a retrospective review of all patients who underwent appendectomy at the Division of General Surgery of Civil Hospital of Ragusa, Italy, between May 2008 and May 2012. The study was performed in accordance to the ethical principles of the Declaration of Helsinki and has been approved by the Ethics committee of ASP of Ragusa (local health authority). The study included 230 patients with diagnosis of acute appendicitis obtained by clinical assessment and confirmed by laboratory blood tests and imaging (US or computed tomography) when deemed necessary. Patients younger than 12 years and patients with preoperative clinical evidence of bowel perforation were excluded. For each patient the age, gender, American Society of Anesthesiologist (ASA) risk score, duration of symptoms, white blood cell (WBC) value upon admission, and previous abdominal surgeries were recorded. Depending on the intra-operative evaluation, the cases of acute appendicitis were divided into uncomplicated or complicated if phlegmon with peri-appendiceal abscess, gangrene, or perforation were noted during surgery. According to the surgical approach performed, the patients were divided into two cohorts, LA group and OA group. Mean operative time, intra-operative and postoperative complications, mean duration of postoperative ileus, and average length of hospital stay were recorded for each group. The total hospital costs were calculated as a mean for each group. The cost for each patient was assessed taking into account the length of time that the operating room was used for surgery, the cost of the material used during the surgery, and the cost of the hospital stay. Cases of conversion from laparoscopic to open appendectomy were included in the LA group.

All laparoscopic procedures were performed by two experienced laparoscopic surgeons (VM, VA), who approached all cases laparoscopically according to a standardized technique that involves the use of three trocars, two 10 mm and one 5 mm. After bipolar coagulation and division with scissors of the mesoappendix, two endoscopic loop ligatures were applied at the base of the appendix; the appendix was then divided and extracted with a bag. All the appendectomies of the open group were performed with McBurney’s incision by other surgeons who preferred this approach a priori. After ligation and division with scissors of the mesoappendix, the base of appendix was ligated with an absorbable tie and the appendix was divided with a scalpel. The appendiceal stump was inverted within the lumen of the cecum using a purse-string suture. Abdominal incision was extended when deemed necessary by the surgeon. Selection bias was minimized because the choice of the surgical approach was not determined by the characteristics of the patient.

The results were analyzed using the t Student test for comparison of data measured as quantitative variables which were age, duration of symptoms, white blood cell count value at the time of hospitalization, operative time, duration of postoperative ileus, length of hospital stay, and total hospital costs. The statistical significance of difference between the categorical variables such as gender, ASA risk score, previous abdominal surgery, uncomplicated and complicated appendicitis, and postoperative complications was calculated using the Chi-square test with Yates correction and Fisher exact test, when appropriate. A p Value < 0.05 was considered statistically significant. Statistical analysis were performed with SPSS computer software (SPSS 21 for MacOS, SPSS Inc., Chicago, IL, USA).

## Results

A laparoscopic approach was performed in 139 patients, an open approach in 91 patients. The two groups were comparable for demographic data (age, gender), comorbidities (ASA risk score), previous abdominal surgery, and clinical severity of the disease quantified by duration of symptoms, WBC value upon admission, and rate of complicated appendicitis (Table 1). In both groups, no mortality was reported, no early readmission (within 30 days of surgery) or re-exploration were needed. The mean operative time was 52.2 min (range 20 min - 155 min) for the LA group and 49.3 min (range 20 min - 110 min) for the OA group, with a no statistically significant difference (p Value 0.476) (Table 2). Two conversions (1.4%) from laparoscopic to open appendectomy were needed. The reasons for conversion were the presence of extensive visceral adhesions in a patient and the presence of a fecal peritonitis secondary to a perforated appendicitis in another patient. Regarding the intra-operative complications, a bladder lesion was detected in the OA group. The total postoperative morbidity rate was 6.9% (16 cases). A postoperative complication occurred in 4 patients (2.9%) of the LA group and in 12 patients (13.2%) of the OA group, with a statistically significant difference in favor of the LA group (p Value 0.0061). (Table 2) There were five wound infections (all in the OA group), 3 intra-abdominal abscesses, all treated conservatively (2 in the OA and 1 in the LA group), 2 cases of prolonged diarrhea (1 in the OA group and 1 in the LA group), 4 cases of prolonged ileus (1 in the LA group, 3 in the OA group), 1 case of pleurisy (OA group), 1 case of urinary tract infection (group LA) (Table 3). There were significant less wound infections in the LA group (p Value 0.009). There was no statistically significant difference between the two groups in the rate of intra-abdominal abscess (p Value 0.563), prolonged diarrhea (p Value 1.000), prolonged ileus (p Value 0.303), pleurisy (p Value 0.395), and urinary tract infection (p Value 1.000). (Table 3) The mean duration of postoperative ileus was 1.2 days (range 1 – 4) in the LA group and 1.4 days (range 1 – 5) in the OA group, with a statistically significant difference of -0.2 day (p Value 0.024). Mean hospital stay was found to be significant shorter (p Value 0.011) in the LA group, 2.75 days (range 1 – 8), compared to the OA group, 3.87 days (range 1 – 19). The average total hospital costs were respectively Euro 2282 (range 1750 – 4912) for the LA group and Euro 2337 (range 1212 – 9947) for the OA group, with a difference of -55 Euro, which was not found to be statistically significant (p Value = 0.812) (Table 2).

**Table 1:** Demographic and clinical characteristics of patients

	**LA group ( *n. 139 *)**	**OA group (n. *91*)**	**p value**
Mean age (years)	28.9 (12 – 73)	31.3 (12 – 48)	0.484
Gender			0.796
Female	62 (44.6%)	43 (47.3%)	
Male	77 (55.4%)	48 (52.7%)	
Previous abdominal operations	11 (7.9)	5 (5.5%)	0.659
WBC count (n x 10^3^/mL)	12.3 (4.8 – 29.8)	13.2 (4.9 – 22.6)	0.812
Duration of symptoms (days)	2.06 (1 – 21)	1.81 (1 – 11)	0.331
ASA risk score			
2	48 (34.5%)	28 (30.7%)	0.653
3	6 (4.3%)	5 (5.4%)	0.925
Complicated appendicitis	39 (28%)	24 (26.3%)	0.897
Abscess	15	8	
Gangrenous	17	12	
Perforated	7	4	

LA: laparoscopic appendectomy; OA: open appendectomy; WBC: white blood cell; ASA: American Society of Anesthesiologist.

**Table 2:** Outcomes

	**LA group**	**OA group**	**Difference**	**95% CI**	**p value**
Mean operative time (min)	52.2	49.3	2.9	-3.08 to 9.09	0.476
Post-op complications rate	2.9%	13.2%	-10.3	-18.9 to -3.3	0.006
Duration of post-op ileus (days)	1.2	1.4	-0.2	0.33 to -0.03	0.024
Mean hospital stay (days)	2.75	3.87	-1.12	-1.25 to 0.33	0.011
Mean total cost (Euro)	2282	2337	-55	-411.24 to 334.34	0.812

CI: confidence interval; post-op: postoperative.

**Table 3:** Post-operative complications

	**LA group**	**OA group**	**p value**
Wound infection	0	5	0.009
Intra-abdominal abscess	1	2	0.563
Diarrhea	1	1	1.000
Prolonged ileus	1	3	0.303
Pleurisy	0	1	0.395
Urinary tract infection	1	0	1.000

## Discussion

LA has become the approach of choice by many surgeons in the treatment of both simple and complicated cases of acute appendicitis. The rate of LA between 1998 and 2008 increased from 20.6% to 70.8%, becoming the prevalent approach to treat acute appendicitis since 2005 [2]. In addition to the clinical benefits described in several studies, the laparoscopic approach allows a full exploration of the peritoneal cavity [11], thus representing an important diagnostic tool in case there is only suspicion of acute appendicitis. Several diseases such as pelvic inflammatory disease, endometriosis, ovarian cysts, ectopic pregnancy, cholecystitis and colonic perforation may mimic appendicitis [12]. In young fertile women 50% of the surgical procedures performed for suspected acute appendicitis turn out not to be acute appendicitis, unless proper imaging was performed [13]. A definite diagnosis is obtained in 96% of patients undergoing LA compared with 72% of those undergoing open procedures.

The LA has been proposed as the preferred technique in obese patients with suspected acute appendicitis, including the elderly patients [14]. In these patients the laparoscopic approach is associated with reduced hospital stay, less postoperative morbidity, and lower cost compared to open approach.

Despite the obvious advantages described, the advantage of LA still remains a matter of debate because of concerns about possible longer operative time, higher rate of postoperative intra-abdominal abscesses, and higher costs compared to OA. Because of all of the above, the open approach appears to be still widely used in clinical practice.

In our study the mean operative time was similar for the two different procedures, with a difference of 2.9 min in favor of OA group that was not found to be statistically significant. Probably, as suggested in other studies [15,16], this finding is related to the experience of the surgeon who performs the laparoscopic procedure, especially in the case of complicated appendicitis, in which the laparoscopic dissection can be technically more complex and therefore time-consuming. A worldwide spread of training in laparoscopic techniques lead to a significant reduction in difference of operative time compared to open procedures after 2000, as evidenced by several meta-analyses [17,18].

The present study confirmed a significant lower incidence of postoperative complications in the cohort of patients treated by laparoscopic approach. This result is consistent with the data shown in a recent meta-analysis [8], which reported a lower rate of postoperative complications, especially surgical wound infection rate, after LA.

Although the infection of the surgical wound is not per se a life-threatening condition, it worsens the quality of life in the early postoperative period and prolongs the recovery time. The reduction of wound infection rate is a significant advantage of LA [18]. The extraction of specimen with a bag and through a trocar port rather than directly through the surgical wound as in open procedures, can explain this reduction in incidence. Moreover, the smaller size of the laparoscopic incisions reduces the probability of infection, especially in obese patients.

The occurrence of an intra-abdominal abscess after appendectomy represents a potentially life-threatening event. Several meta-analyzes of randomized controlled trials (RCT) published in recent years [8,17-19] have shown an increased risk of intra-abdominal abscesses after LA. It has been suggested that this complication may be related mainly to an improper laparoscopic technique, such as an aggressive handling of infected appendix or an excessive use of irrigation fluids, which could lead to significant contamination of the peritoneal cavity [10,20]. However, the most recent meta-analysis of RCT published [9] shows a low incidence of intra-abdominal infections, with no significant difference between the laparoscopic and the open approach. In our opinion, this finding might be due to an increase in the laparoscopic skills of the surgeons performing the procedure as previously suggested by some authors [21,22]. In our study, the intra-abdominal abscess rate was very low (1.3%) and there was no significant difference between the two different approaches. These findings are probably related to exclusion from our study of patients with clinical signs of perforated appendix and also to the high laparoscopic skills of surgeons that carried out the LA.

In our study, the difference of duration of postoperative ileus between the two different approaches was statistically significant. Recovery of the bowel function was faster in the LA group (1.2 days vs 1.4 days, p Value 0.02). Factors such as reduced manipulation of the ileum and the cecum in the hands of a skilled surgeon, as well as a minor abdominal trauma and less pain due to the smaller extension of the incision of the trocars, and an early postoperative mobilization of the patient can be invoked to explain these data [9,18,23,24].

In our experience, the length of the hospital stay was about 1 day shorter in the LA group than in the OA group (p value 0.011). This result is comparable to the results of the meta-analysis by Wei et al. [9], which also showed that patients undergoing LA return earlier to work and to normal daily activities. The reduction in length of hospital stay seen in the LA group has a direct impact on costs. Although the cost of the laparoscopic approach can be higher than cost of open approach because of the use of disposable instruments and ports, the difference in total costs between the two procedures is decreased by the shorter length of stay experienced by patients who underwent LA [25].

Our analysis showed a no significant difference in terms of total hospital costs between the two procedures. Nakhamiyayev [16] and Varela [14] reported that the total hospital costs was comparable between the two procedures or even lower for the laparoscopic group when the subgroup of obese patients was analyzed. Wei et al. [9] in their meta-analysis including 8 RCTs [26-33] performed an analysis of the costs across different countries and age groups using the hospital cost ratio to compare the total cost of LA and OA. The total hospital costs for LA were higher by 11% when compared to OA, but the difference was found to be no statistically significant. However, these data are in contrast with those recently published by McGrath et al. [2], who compared the costs between LA and OA in 2,887,823 patients undergoing surgery in the period between 1998 and 2008. Despite the LA was associated with a lower incidence of complications and a shorter hospital stay compared to OA, it was significantly more expensive in both simple and complex cases. The cases of conversion were associated with a higher postoperative morbidity and higher costs. The cost of OA was lower when postoperative complications occurred, but the cost difference between the two procedures was no significant when there was at least one complication in complex cases, and more than one complication in simple cases. The authors believe that the costs of laparoscopy remain still higher because of the increased operative time and the higher cost of laparoscopic instruments. However, the study did not include the analysis of the complications occurred after discharge and the rate of readmission, and instead included cases performed by surgeons with no laparoscopic training.

A limitation of our study is its retrospective nature. It also does not take into account the long-term complications and their effect on health care costs. The two groups of patients analyzed, however are homogeneous. The bias on the choice of treatment is minimized by the fact that the operating surgeons are different for each group and the choice of treatment did not occur according to the characteristics of the patient but to operator preference for one of the two techniques. Ours is a small district hospital and each operator had 10 years expertise for the surgical procedure of his choice.

## Conclusions

In our study we compared the outcomes between laparoscopic and open appendectomy for treatment of acute appendicitis performed in a district hospital. Our results show that laparoscopic appendectomy is associated with fewer postoperative complications, mainly due to a lower incidence of wound infections, with a rate of intra-abdominal abscess similar to open appendectomy. The operative time of laparoscopic treatment is comparable with that of open treatment, but length of hospital stay is shorter after laparoscopy. These findings have a direct impact on total costs, which are similar for the two procedures. Therefore, laparoscopic appendectomy can be recommended as preferred approach for treatment of acute appendicitis.

### Consent

Written informed consent was obtained from the patient or parents in the case of patient under 18, for the publication of this report and any accompanying images.

## Abbreviations

ASA: American Society of Anesthesiologist; LA: Laparoscopic appendectomy; OA: Open appendectomy; RCT: Randomized clinical trial; US: Ultrasonography; WBC: White blood cell.

## Competing interests

Goffredo Arena is a surgical consultant for Covidien, Canada. All authors declare that they have no competing interests.

## Authors’ contributions

VM and VA conceived and coordinate the study and participated in statistical analysis. VM and AL drafted the manuscript. AL collected patients’ data and performed statistical analysis. BDS, MA and GA participated in prepared and drafting the manuscript. All authors read and approved the final manuscript.

## Authors’ information

MV is Professor of Surgery at University of Catania, Italy; he was Chief of the Division of General Surgery at the Civil Hospital of Ragusa, Italy, until May 2013.

## Pre-publication history

The pre-publication history for this paper can be accessed here:

http://www.biomedcentral.com/1471-2482/14/14/prepub
